# Recurrence of Vogt-Koyanagi-Harada Disease as Immune-Related Adverse Events Induced by an Immune Checkpoint Inhibitor

**DOI:** 10.7759/cureus.114900

**Published:** 2026-08-20

**Authors:** Atsuki Fukushima, Hitoshi Tabuchi

**Affiliations:** 1 Ophthalmology, Tsukazaki Hospital, Himeji, JPN; 2 Ophthalmology, Hiroshima University, Hiroshima, JPN

**Keywords:** immune checkpoint inhibitor, immune-related adverse events, optical coherent tomography, uveitis, vogt-koyanagi-harada disease

## Abstract

We describe a case involving recurrence of Vogt-Koyanagi-Harada (VKH) disease induced by treatment with the immune checkpoint inhibitor (ICI) pembrolizumab (anti-programmed death (PD)-1 antibody). The patient was a 59-year-old woman who presented with decreased vision in both eyes. She was referred to our hospital. In addition to anterior chamber inflammation, thickening of the choroid, folds in the retinal pigment epithelium (RPE) layer, and multiple serous retinal detachments were observed. Cerebrospinal fluid analysis revealed a significant increase in mononuclear cells, leading to a diagnosis of VKH disease. She received an intravenous infusion of 1,000 mg of methylprednisolone for three days; thereafter, she was started on 40 mg of oral prednisolone, which was gradually tapered off, and treatment was completed 18 months after the onset.

One year later, the patient underwent tumor resection for endometrial cancer and was subsequently treated with the ICI anti-PD-1 antibody (pembrolizumab). She began noticing a decline in her vision, and a recurrence of VKH disease was noted. Thickening of the choroid, folds in the RPE layer, and serous retinal detachments were again observed in both eyes, leading to a diagnosis of recurrent VKH disease. While continuing pembrolizumab, the patient resumed oral prednisolone at 30 mg. As improvement was observed, the dose of prednisolone was gradually tapered; however, anterior chamber inflammation, thickening of the choroid, and RPE folds were subsequently noted, leading to a diagnosis of another recurrence. When administering an ICI to patients with a history of VKH disease, an ophthalmology consultation should be recommended both before and after initiating treatment.

## Introduction

Immune checkpoint inhibitors (ICIs) work to control cancer by blocking immunosuppressive signals and thereby activating T cells to attack tumors [1]. ICIs have been shown not only to activate tumor-specific T cells but also to activate various T-cell populations, particularly autoreactive T cells, which are normally present at very low frequencies [2]. As a result, various autoimmune and inflammatory diseases may develop in different organs as immune-related adverse events (irAEs). IrAE-related diseases in ophthalmology are relatively rare [3]. This may be partly attributable to the fact that the eye is an immunologically privileged organ [4]. Although rare, irAE-related ocular diseases can affect both the anterior segment and the retina and may significantly impair visual function [1]. Regarding anterior segment inflammation, various ocular conditions, including dry eye, conjunctivitis, and keratitis, have been reported. On the other hand, among diseases presenting with retinal lesions, uveitis has been reported sporadically, particularly cases resembling Vogt-Koyanagi-Harada (VKH) disease [5-10]. We report a case of a patient with VKH disease who achieved remission with systemic steroid therapy and subsequently discontinued treatment, but later developed endometrial cancer and underwent ICI therapy, and subsequently experienced a recurrence of VKH disease.

## Case presentation

**Figure 1:**
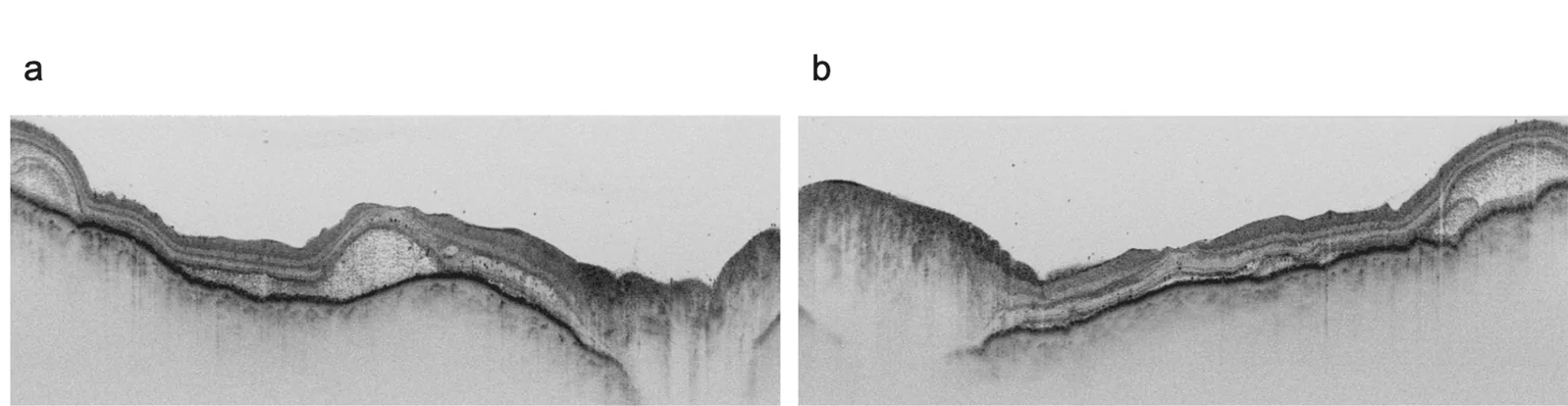
Swept-source OCT findings at the initial visit a: right eye. b: left eye. Thickening of the choroid, folds in the RPE cell layer, and multiple serous retinal detachments were observed in both eyes OCT: optical coherence tomography; RPE: retinal pigment epithelium

A 59-year-old woman presented with a one-week history of decreased vision in both eyes and was referred to our hospital. At the initial visit, corrected visual acuity was 0.5 in the right eye and 0.8 in the left eye. In addition to anterior chamber inflammation, thickening of the choroid, folds in the retinal pigment epithelium (RPE) cell layer, and multiple serous retinal detachments were observed in both eyes (Figure 1).

**Figure 2:**
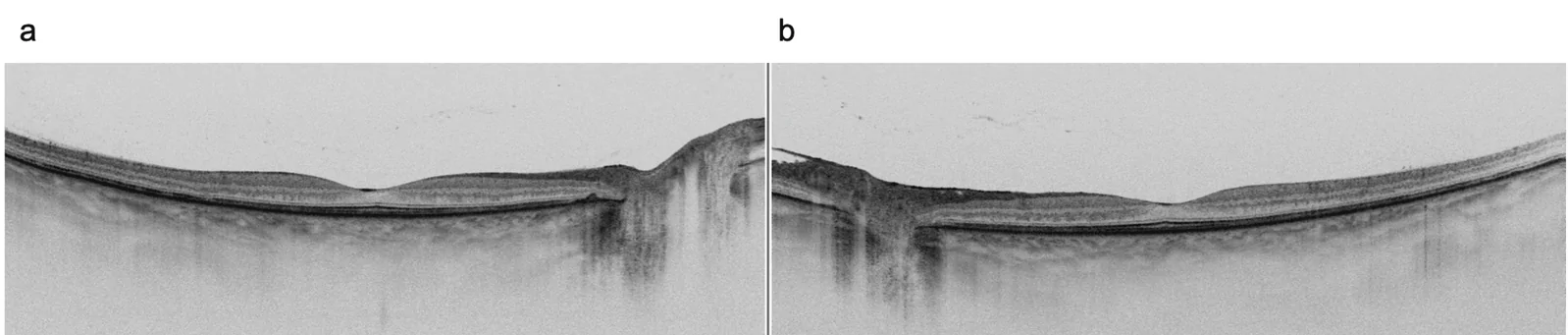
Swept-source OCT findings at the end of prednisolone treatment a: right eye. b: left eye. No abnormalities were found in either eye OCT: optical coherence tomography

A cerebrospinal fluid examination was performed, revealing a significant increase in mononuclear cells, which led to a diagnosis of VKH disease. Hair loss and hair whitening were observed; however, neither skin depigmentation nor otologic symptoms were detected. The patient then received an intravenous infusion of 1,000 mg of methylprednisolone for three days, followed by oral prednisolone 40 mg, which was gradually tapered. No immunomodulatory drugs were prescribed throughout the treatment period. Anterior chamber inflammation and serous retinal detachment resolved in both eyes (Figure 2), and 18 months later, treatment was temporarily discontinued.

**Figure 3:**
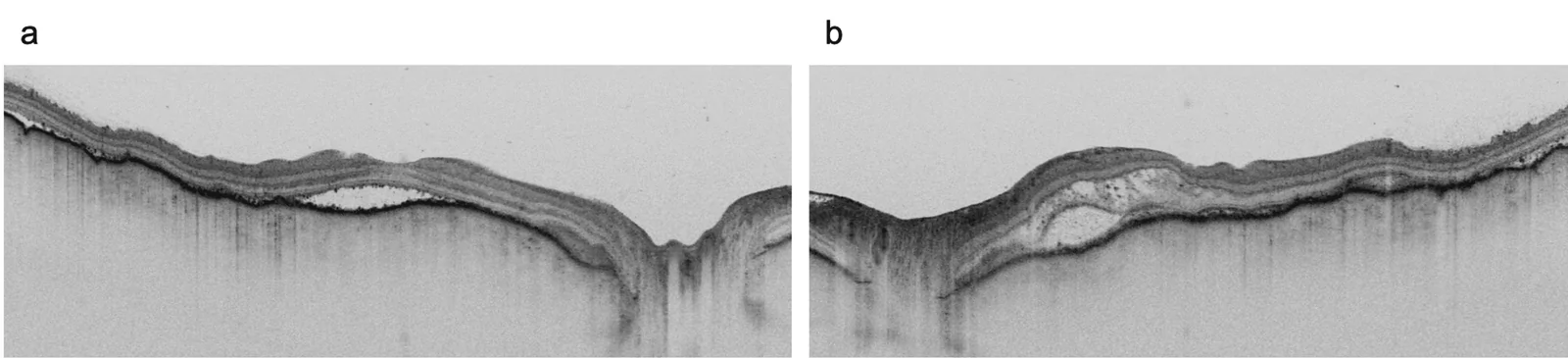
Swept-source OCT findings at the first recurrence a: right eye. b: left eye. Thickening of the choroid, folds in the RPE cell layer, and multiple serous retinal detachments were observed in both eyes OCT: optical coherence tomography; RPE: retinal pigment epithelium

At the time of discontinuing oral prednisolone, corrected visual acuity was 1.2 in both eyes. One year after prednisolone discontinuation, the patient underwent tumor resection for endometrial cancer. Subsequently, she received the anti-programmed death (PD)-1 antibody pembrolizumab every three weeks. No other medication was prescribed for endometrial cancer. She began noticing decreased vision two months after tumor resection. One week later, she was referred to our hospital, and her corrected visual acuity was 0.8 in the right eye and 0.3 in the left eye. Choroidal thickening, folds in the RPE cell layer, and serous retinal detachment were again observed in both eyes (Figure 3), leading to a diagnosis of recurrent VKH disease. However, indocyanine green fluorescence angiography and fluorescein fundus angiography were not performed.

**Figure 4:**
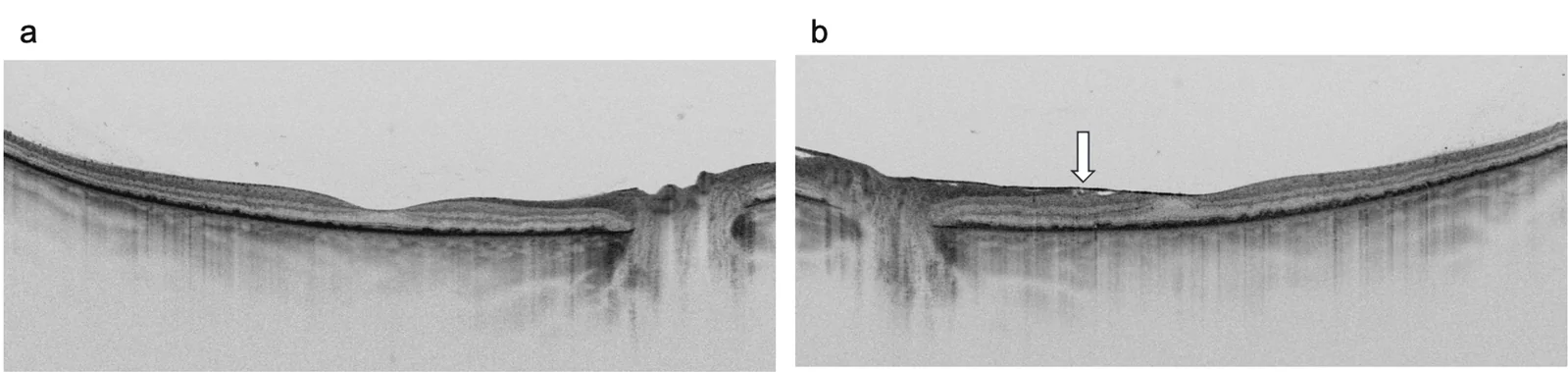
Swept-source OCT findings after the treatment for the first recurrence a: right eye. b: left eye. Folds in the RPE cell layer and serous retinal detachments were not found in either eye. However, thickening of the choroid was found in both eyes. ERM was detected in the left eye (white arrow) OCT: optical coherence tomography; ERM: epiretinal membrane

While continuing pembrolizumab, oral prednisolone was restarted at a dose of 30 mg. One month later, the serous retinal detachments had resolved; however, choroidal thickening persisted (Figure 4). Additionally, an epiretinal membrane (ERM) was detected in the left eye (Figure 4).

**Figure 5:**
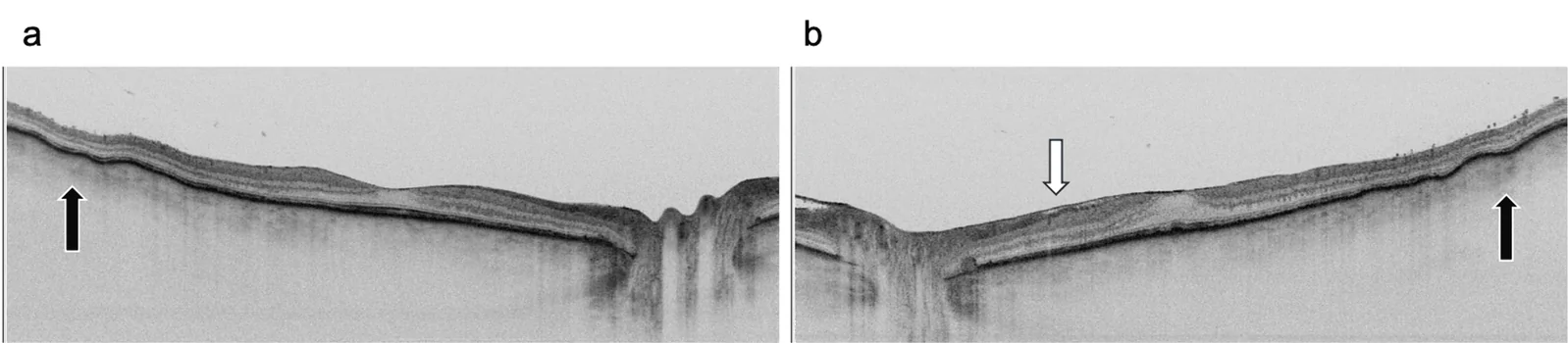
Swept-source OCT findings at the second recurrence a: right eye. b: left eye. In addition to thickening of the choroid, folds in the RPE cell layer (black arrows) were observed in both eyes. ERM was detected in the left eye (white arrow) OCT: optical coherence tomography; RPE: retinal pigment epithelium; ERM: epiretinal membrane

Thereafter, the prednisolone dose was gradually tapered. Five months after starting prednisolone treatment, the patient was receiving 10 mg of prednisolone daily. Eight weeks later, anterior chamber inflammation, choroidal thickening, and RPE folds were again observed in both eyes, although serous retinal detachment was not observed (Figure 5). ERM was detected in the left eye (Figure 5).

Pembrolizumab was continued, and prednisolone was maintained at a dose of 10 mg without further tapering.

## Discussion

irAE refers to immune abnormalities, primarily autoimmune reactions, that occur following ICI administration. irAEs can cause damage to various organs, and their course and response to treatment vary widely [5]. The most common irAEs are diarrhea/colitis, fatigue, and maculopapular rash [3]. Less frequently, myocarditis, uveitis, and aseptic meningitis have also been reported [3]. According to a study on ophthalmic irAEs by 29 panelists from 25 academic medical centers, ophthalmic irAEs are recommended to be classified into two groups: optic nerve and orbital disorders, and uveitis and ocular surface disorders [5]. irAE-associated uveitis can also be classified according to anatomical location (anterior, intermediate, posterior, or panuveitis) and subtype (VKH-like or sarcoidosis-like). Treatment guidelines tailored to each subtype have been proposed. For example, for VKH-like syndrome (VKHLS), steroid-based treatment is recommended according to the clinical presentation and severity, similar to the treatment for VKH [5].

It has been reported that approximately 10% of irAE uveitis cases are VKHLS [6-8]. In reports from Japan, six out of 15 cases of irAE uveitis were VKHLS [9], and another report indicated that five out of nine cases were VKHLS [10]. Since there are racial differences in the incidence of VKH, with a higher incidence among Asians [11], the incidence of VKHLS is also thought to be high among Japanese individuals. Furthermore, in the former report [9], cases that developed in patients with malignant melanoma following administration of PD-1/PD-1 ligand inhibitors accounted for half of all VKHLS cases; therefore, it is believed that, similar to VKH, an immune response targeting melanocytes plays a key role in the onset of VKHLS. It was reported that, in addition to PD-1/PD-1 ligand inhibitors, the anti-cytotoxic T-lymphocyte antigen-4 (CTLA-4) antibody ipilimumab can induce ocular and orbital inflammation [12]. Regardless of the type of ICI administered, ocular and orbital irAEs should be evaluated when ICIs are administered.

It has been reported that irAEs are more likely to occur in patients with a history of autoimmune diseases. Patients with a history of autoimmune diseases have been reported to have an odds ratio of 2.52 for developing irAEs compared with those without such a history [13]. Furthermore, a meta-analysis of 23,897 cancer patients with a history of autoimmune disease reported that 61% developed irAEs, and 36% experienced a relapse of their previous autoimmune disease [14]. These reports clearly indicate that patients with a history of autoimmune disease are more likely to develop irAEs and are also more prone to relapse of their pre-existing autoimmune disease. Thus, the recurrent uveitis in this patient could be considered a relapse of VKH.

To the best of our knowledge, there has been only one reported case of VKHLS developing as an irAE in a patient with VKH [15]. As described in previous papers [6-10], the ophthalmic findings of VKHLS and VKH, including optical coherence tomography (OCT) findings, are remarkably similar. Since the onset of VKH in that case report occurred more than 25 years before the development of VKHLS [15], this demonstrates that memory autoreactive T cells can persist for prolonged periods. Therefore, given that our case occurred within two years of the onset of VKH, the likelihood of developing VKHLS would naturally be considered high.

## Conclusions

When administering ICIs to patients, it is essential to obtain a history of autoimmune ocular diseases, such as VKH. When administering ICIs to patients with a history of VKH, an ophthalmologic consultation should be recommended both before and after treatment initiation, even if the VKH is in remission.
